# Information Resource Description: Creating and Managing Metadata. Second Edition

**DOI:** 10.29173/jchla29501

**Published:** 2021-04-02

**Authors:** Vanessa Kitchin

**Affiliations:** Librarian & Instructor: Medicine & Dentistry, University of British Columbia, Vancouver, BC, Canada

Hider P. **Information Resource Description: Creating and Managing Metadata. Second Edition**. United States: ALA: Neal Schuman; 2018. Softcover: 277 p. ISBN: 978-0-8389-1836-4. Price: USD $84.99. Available from: https://www.alastore.ala.org/content/information-resource-description-creating-and-managing-metadata-second-edition

Philip Hider’s second edition of *Information Resource Description* provides a broad yet detailed sweep of metadata best practices and applications. It is clearly organized and steeped with references and links to an incredibly robust amount of resources. I am confident in proclaiming it a bible for any information professional who is new to metadata application or who wants to improve their understanding of the landscape and trends in the complex world of seemingly infinite metadata elements. While many library associations have seminal works that are discipline- specific, such as the Music Library Association’s guide for sound and audiovisual archives (Jenn Riley’s *Glossary of Metadata Standards*, for example), Hider’s sweeping text provides a foundational resource for those new to this aspect of librarianship and one that can be applied to many subject areas. It expands on the discipline-specific texts by incorporating an element of quality improvement and systems management for which librarian expertise is required and able to be widely applied.

Philip Hider is Head of the School of Information Studies at Charles Sturt University, Australia. He “has worked, taught and researched in the field of information organization in the UK, Singapore and Australia. He holds a PhD from City University, London and was made a fellow of CILIP in 2004” [ALA, n.d.]. His impressive educational and work history is clearly evident in the breadth of knowledge and writing ability exemplified in the book. A key highlight of Hider’s second edition is the chapter on information resource attributes. Many librarians and research data management experts are fundamentally aware of the value of good metadata practices but Hider’s overview and categorization of complex concepts expounds the body of knowledge more conceptually. Practices that Hider describes include relevance criteria, service convergence, algorithmic contributions, and aboutness, providing a comprehensive underpinning for metadata attributes. One of many strengths of this work includes an aboutness concept map that aptly probes with the following metadata standardized descriptors: ‘may be distinguished between; determination may involve; is sometimes considered; is constructed of the; towards subjective perspective related to; the post-modern perspective, etc.’ all as a means for creating information resource description through an interpretive process. Hider further identifies the need for human description versus the contribution of algorithmic applications for description and makes this relevant to the reader. The overview this chapter provides lays the groundwork for identifying the attributes most users or engagers of metadata want to know about.

While the text employs exemplary use of tables and diagrams, the only critique of this work is that more visual aids such as charts, tables, diagrams and concept maps used as examples of the technical and theoretical elements of information resource description could further inform and develop a novice’s understanding.

This book serves as both a practical tool and philosophical conjuring of the meaning of information organization, description and retrieval. As Hider notes: “things have been arranged by people for many thousands of years, and information resources have been arranged for as long as there have been collections of them.” The ‘Tools and Systems’ chapter looks into the new manifestations required for accurate metadata arrangement. Hider attests that arrangement must adhere to conventions and standards and must also specify order in ways to make accessibility streamlined. Of particular use to medical librarians are the delineations of vocabularies within databases and online information retrieval systems that Hider outlines. The searches medical librarians create and execute are logical puzzles that distill conceptual research questions into systematized means of data retrieval. Hider provides a clear explanation of the modes of description with a helpful user interface and applications diagram on page 128. This diagram illustrates schema design as foundational to ontology, roles and query, logic, proof, and trust with signature and encryption as adjacent elements. The concise yet effective description of standards and the way they inform or stack within the semantic web allow for an additionally historical perspective on resource description management and how Dublin Core, XML schema definition and document type definition evolved. Hider also sweeps through the historical record as it relates to guidelines for library catalogues, the first having been issued by France’s revolutionary government in 1791.

At once a handbook, tool book, bibliography and resource guide, the second edition of *Information Resource Description* illustrates standards and complicated aspects of metadata concepts such as entities and relationships in an accessible way. The chapter that discusses the future of metadata and information resource description provides yet another foundational overview and discussion of the prospects for different metadata approaches. Of particular note, and one that may interest metadata instructors, is the way Hider delineates the three general ways to approach access to information: one which bypasses metadata altogether; one that employs the Web 2.0 approach, where end-users and contributors provide the metadata; and finally, the traditional approach, in which resources are organized and described by information professionals which they deem most effective. Hider leaves the reader to ponder whether quality metadata will be valued in the systems of the future, another theoretical underpinning relevant to the future of our profession and those enrolled in information science graduate studies.

The purpose of the second edition remains the same as the first: to provide a foundation and broad overview of information description while also contributing a robust list of resources and elementary understanding that prepare for more in-depth study. The structure remains the same as the first edition, with updates that include the addition of BIBFRAME and the rewriting of certain sections for clarity of concept. While each chapter has an enviable list of resources, the author also provides the reader with a broader and more general bibliography at the end.

Recent positions in health sciences librarianship have encompassed an element of metadata expertise and include roles such as Metadata Transformation Librarian and Metadata and Digital Initiatives Librarian. Information description informs the traversal of the medical literature which results in effective and sound systematic review methodological rigour. Hider’s text provides a fulsome understanding of metadata standards, how they differ, how they are employed, and how vocabularies shape our access to the full spectrum of information about a topic. It is an accessible, well-written and almost conversational overview of information resources description that leaves the reader with an understanding of the elemental nature of metadata, its origins and ponderings on its future.
